# Maintaining the Balance: Regulation of NK Cell Activity

**DOI:** 10.3390/cells13171464

**Published:** 2024-08-31

**Authors:** Vanna Imširović, Felix M. Wensveen, Bojan Polić, Vedrana Jelenčić

**Affiliations:** Department of Histology and Embryology, Faculty of Medicine, University of Rijeka, 51000 Rijeka, Croatia

**Keywords:** NK cells, activation, inhibitory receptors, activating receptors

## Abstract

Natural Killer (NK) cells, integral components of the innate immune system, play a crucial role in the protection against intracellular threats. Their cytotoxic power requires that activation is tightly controlled, and in this, they take a unique position within the immune system. Rather than depending on the engagement of a single activating receptor, their activation involves a delicate balance between inhibitory and activating signals mediated through an array of surface molecules. Only when this cumulative balance surpasses a specific threshold do NK cells initiate their activity. Remarkably, the activation threshold of NK cells remains robust even when cells express vastly different repertoires of inhibitory and activating receptors. These threshold values seem to be influenced by NK cell interactions with their environment during development and after release from the bone marrow. Understanding how NK cells integrate this intricate pattern of stimuli is an ongoing area of research, particularly relevant for cellular therapies seeking to harness the anti-cancer potential of these cells by modifying surface receptor expression. In this review, we will explore some of the current dogmas regarding NK cell activation and discuss recent literature addressing advances in our understanding of this field.

## 1. Introduction

NK cells belong to the innate lymphoid cell (ILC) family. Unlike B and T cells, which rely on the recombinant activating genes (RAG) for their development, NK cells develop independently of RAGs and are not regulated by antigen-specific receptors [1]. ILCs are categorized into three subsets based on the cytokines and transcription factors they express: group 1 ILCs (ILC1s), group 2 ILCs (ILC2s), and group 3 ILCs (ILC3s). Group 1 ILCs further divide into classical NK cells and ILC1s [2]. Both NK cells and ILC1s can secrete interferon-γ (IFNγ) and tumor necrosis factor (TNF). However, NK cells stand out due to their potent cytolytic functions. Unlike other ILCs, NK cells are circulating cells, while ILCs primarily reside in tissues [3,4,5].

Defining ILC1s and NK cells can be challenging, especially across different tissues and inflammatory conditions. Single-cell analysis has revealed significant heterogeneity within ILC1 subsets. One main distinction is Eomesodermin (EOMES) dependency: NK cells require EOMES for development and maturation, while ILC1s do not. Additionally, murine NK cells express the CD49b marker, whereas ILC1s express markers such as CD103, CD49a, and CXCR6 [1]. It is important to note that the expression of ILC1-specific markers can vary depending on the tissue being analyzed.

In human peripheral blood, NK (natural killer) cells are identified by the presence of the CD56 marker and the absence of the CD3 marker. Human NK cells are further classified based on CD56 expression into two subsets: CD56^dim^ and CD56^bright^. Most NK cells in the spleen and peripheral blood are CD56^dim^ CD16^+^ and express perforin. The CD56^dim^ subset is more cytotoxic and produces IFNγ to a lesser extent, whereas the CD56^bright^ CD16^−^ subset primarily produces cytokines [6].

**Figure 1:**
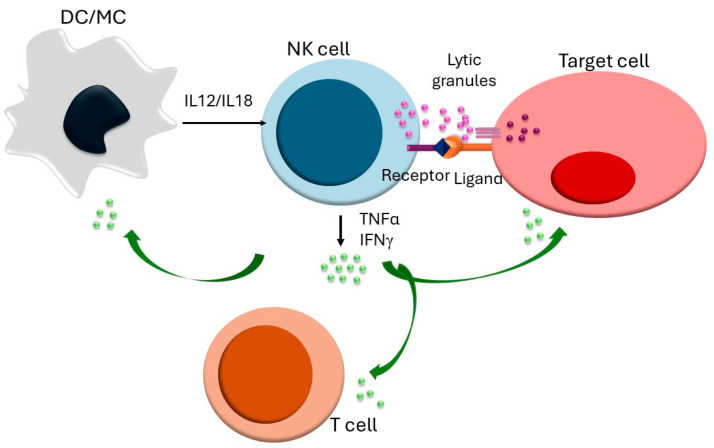
NK cell activation and immune regulation: The binding of an activating receptor to its ligand causes a shift in the balance between activating and inhibitory receptors, resulting in NK cell activation. This activation is further enhanced by the cytokines IL-12 and IL-18, which are produced by dendritic cells and macrophages. Once activated, NK cells can eliminate target cells through the release of lytic granules containing granzyme B, which induce target cell death. Moreover, NK cells secrete cytokines such as IFN-γ and TNF-α, which help modulate the functions of other innate and adaptive immune cells.

NK cells are crucial components of the innate immune response, playing a vital role in early responses to tumors, viral infections, and organ transplants [7]. They possess cytotoxic capabilities and can lyse target cells using various mechanisms, including cytolytic granules containing granzyme and perforin, as well as inducing apoptosis via TRAIL and FASL. Both pathways contribute to a process known as serial killing, where initial kills are mediated by granzyme B and later by FASL [8,9,10]. Additionally, NK cells mediate antibody-dependent cell cytotoxicity (ADCC) [11]. Beyond their cytotoxic functions, NK cells secrete a range of cytokines, such as IFNγ, granulocyte-macrophage colony-stimulating factor (GM-CSF), and TNFα, which modulate the functions of other innate and adaptive immune cells (Figure 1) [12].

There are several ways in which NK cells can exert immune regulation. They influence the homeostasis of dendritic cells (DCs) by killing immature DCs and promoting the cross-presentation of antigens from apoptotic target cells to DCs. NK cells also promote DC maturation by secreting IFNγ and TNFα, which, in turn, further activate NK cells through IL-12 and IL-18 [7]. Additionally, NK cells impact T and B cells during inflammation by promoting the priming of Th1 cells via IFNγ secretion and killing activated T cells that lack sufficient classical or non-classical MHC-I molecules [13,14]. Fas-deficient mouse studies have shown that NK cells can suppress autoreactive B lymphocytes, as NK cell depletion increases the severity of autoimmunity [15]. Thus, NK cells protect the host from pathological agents and regulate excessive immune responses.

NK cells play a crucial role in immune regulation, homeostasis, and defense. Therefore, their activity must be tightly controlled to prevent harmful conditions such as inflammation or autoimmune disorders. On their surface, NK cells stochastically express a variety of germline-encoded activating and inhibitory receptors. Although it is well established that activating signals must outweigh inhibitory ones for NK cell activation, this raises an important question: Is this regulatory balance sensitive enough, or are there additional mechanisms required to ensure proper NK cell activation? Furthermore, numerous genetically modified mice have been created that lack the expression of one or several NK cell receptors. While these mice have shown varying degrees of susceptibility to certain pathological conditions, none have exhibited major defects in NK cell function. This observation prompts another question: How do NK cells compensate for these receptor deficiencies? In this review, we will explore these questions and provide an overview of the known compensatory mechanisms in NK cells.

## 2. NK Cell Receptors and Activation

Unlike T and B cells, NK cells lack recombination-dependent antigen receptors but express many germline-encoded activating and inhibitory receptors. The activation of NK cells is governed by a system known as a balance system, wherein cells receive signals from both activating and inhibitory receptors. Depending on the prevailing signal, NK cells either remain inactive or become activated [12,16]. NK cells survey the body in search of “unhealthy” and potentially dangerous cells. Under normal physiological conditions, NK cell activity is inhibited by interaction between inhibitory receptors and self-MHC-I molecules. If these proteins are expressed at sufficient levels, NK cells receive the appropriate inhibitory stimuli to remain inactive.

The first way by which NK cells are believed to be activated is through a lack of MHC-I molecules or a reduction in their expression. MHC-I molecules are typically downregulated upon viral infection such as cytomegalovirus infection (CMV) [17,18] or in some tumors, for example, bladder, breast, colorectal cancers, and melanoma [19,20,21,22,23], to avoid CD8 T cell-mediated control. This event, therefore, signals that something is wrong. MHC-I downregulation results in a relative reduction of inhibitory stimuli, thus shifting the balance towards activation in a process referred to as “missing self” [24,25]. The second way of activation is through activating receptors. Healthy cells do not express ligands for activating receptors or express them at very low levels. Their expression increases after cells undergo stressful conditions such as infection or oncogenic transformation, which is then detected by activating receptors. This type of NK cell recognition and activation is known as “induced self” and an example of it is the interaction between NKG2D and its ligands, such as MICA/MICB in humans or MULT1 in mice [26]. The third way is known as “non-self” recognition and is unique for the Ly49H activating receptor since it recognizes mouse cytomegalovirus-encoded protein m157, expressed on the surface of infected cells [27,28].

Inhibitory and activating NK cell receptors are a complex group of molecules that structurally can be divided into 2 subgroups: The first group is an immunoglobulin (Ig) like receptor superfamily including KIR (Killer Ig-like receptors), LIR (leukocyte Ig receptors) expressed only in humans, and NCR (Natural cytotoxicity receptors) expressed both in human and mice. The second group includes C-type lectin-like receptors (CTLR), including the NKG2 (NKG2A to F) and Ly49 receptor families [16].

### 2.1. Activating Receptors

Activating receptors can provide NK cells with a strong stimulus in the absence of co-stimulation due to the presence of adaptor molecules such as DAP12, DAP10, FcRγ, and CD3ζ that contain immunoreceptor tyrosine-based activating motifs (ITAMs). FcRγ and DAP12 have a single ITAM, while CD3ζ has three ITAMs per chain. Phosphorylated ITAM induces a signaling cascade in the Syk family of kinases, most notably Syk or ZAP-70, resulting in the activation of the cell [29].

Numerous activating receptors adorn the surface of NK cells. As mentioned previously, most of them recognize ligands that are usually not expressed on healthy cells, but their expression increases after cells undergo stressful conditions such as infection or oncogenic transformation. Notable examples are NKG2D, NKp46 (NCR1), and DNAM-1, which play an important role in the control of different pathological conditions. NKG2D (coded by *Klrk1)* is constitutively expressed on all NK cells from the earliest stages of NK cell development in the pre-pro NK cells [30]. Throughout their development, expression of NKG2D increases and stays high at their mature stage [30]. Virus-infected cells upregulate NKG2D ligands, which makes them susceptible to NKG2D-mediated NK cell elimination. Murine cytomegalovirus (MCMV) dedicates several genes to disrupt the expression of NKG2D ligands (m138, m145, m152, and m155), which emphasizes its importance [31,32,33]. The NKG2D receptor also plays an important role in tumor surveillance since many tumors, especially in the early stages of oncogenesis, express NKG2D ligands. It was shown that transfection of resistant tumors with NKG2D ligands makes them susceptible to NK cell-mediated control in vivo and in vitro [34,35]. The importance of this receptor was further illustrated by the fact that *Klrk1*^−/−^ mice rapidly succumbed to NKG2D-ligand-expressing cells upon their transfer [36,37]. Although the cellular ligands for NKp46 are still mostly unknown, the role of NKp46 receptors in tumor immunology, as well as in bacterial and viral infections, is well investigated [38,39,40]. The NKp46 receptor plays an important role in regulating the development of metastasis and lymphoma [39,41]. DNAM-1 (CD226) plays a crucial role in the recognition and elimination of tumor cells by both NK and T cells. In vivo, tumor rejection was notably diminished in mice lacking DNAM-1 expression, particularly in models where the DNAM-1 ligand PVR was expressed on tumor cells [42].

While most ligands for activating receptors are expressed on the cell surface, recent studies have shown that soluble ligands can also activate NK cells. For example, activation with soluble ligands can occur through the NKG2D and NKp44 receptors. Tumors often try to evade NK cell-mediated control by shedding membrane ligands that bind to NKG2D receptors, causing NK cell desensitization. However, Deng et al. demonstrated that shedding the high-affinity NKG2D ligand MULT-1 has the opposite effect, resulting in increased NK cell activation and better tumor control [43].

Additionally, it has been shown that platelet-derived growth factor (PDGF)-DD, secreted by many cancers to support tumor growth and stromal reactions, acts as a ligand for the NKp44 activating receptor. The interaction between the NKp44 receptor and PDGF-DD triggers the secretion of IFN-γ and TNF-α, leading to tumor cell growth arrest [44]. This shows that the mechanism used by tumors to promote their growth and evade the immune system can backfire and enhance NK cell-mediated control.

### 2.2. Inhibitory Receptors

While activating receptors recognize ligands that are pathogen-derived or stress-induced, inhibitory receptors recognize constantly expressed self-proteins, most notably MHC-I. Inhibitory receptors include members of the Ly49 receptor family in mice, killer cell immunoglobulin-like receptors (KIRs) in humans, and CD94/NKG2A receptors. In their cytoplasmic tail, inhibitory receptors contain ITIMs (immunoreceptor tyrosine-based inhibitory motif). MHC-I engagement of inhibitory receptors induces phosphorylation of ITIMs, which recruits the Src-homology 2 (SH2) domain-containing protein tyrosine phosphatases such as SHP-1 and SHP-2 and consequently induces NK cell inhibition [45]. Inhibitory receptors are stochastically expressed on NK cells, and an individual NK cell can simultaneously express multiple inhibitory receptors, even some that do not recognize self-MHC [46]. Inhibitory receptors play an important role in a process called licensing or education that ensures proper NK cell reactivity as well as tolerance toward self [24], which will be addressed later in this review.

To assess the significance of specific activating or inhibitory receptors, numerous genetically modified mice lacking the expression of one or even several receptors were generated. The majority of defects observed in NK cells from these mice were directed toward targets that expressed ligands specific to particular mutated receptors. For instance, NK cells deficient in the NKG2D, NKp46, or DNAM-1 receptor exhibited diminished capability to eliminate target cells expressing corresponding ligands or showed decreased survival in cases where tumors expressed those ligands [36,42,47]. Also, NKp46 deficient mice showed reduced lung NK cell activation and IFNγ production after S. pneumoniae infection [40]. While certain genetically modified mice lacked one or more crucial NK cell receptors, the overall functionality of NK cells in these mice remained intact. Moreover, these mice did not exhibit spontaneous autoimmunity or develop cancers, suggesting the intricate regulation of NK cell activity. This observation also implies that NK cells possess compensatory mechanisms to overcome such deficiencies.

## 3. Regulation of NK Cell Activity

NK cell activation is a tightly regulated interplay between inhibitory and activating signals that cells receive through receptors expressed on their cell surface and/or a variety of cytokines produced by other hematological and nonhematological cells [48]. This tight regulation is required to avoid autoimmune diseases, excessive inflammation, or immune-related damage. Although NK cells have been extensively investigated for almost half a century, this complex process of NK cell activation is still not completely understood. Many things contribute to this complexity, and we will mention some of them in this review.

### 3.1. Regulation of NK Cell Responsiveness during Development

#### 3.1.1. Education through Inhibitory Receptors

For NK cells to become functional, self-specific inhibitory receptors must interact with their ligands. This is a process known as licensing or education, which occurs during NK cell development [49]. This means that NK cells must possess at least one inhibitory receptor specific to self-MHC-I molecules [49]. Evidence for this model comes from experiments performed in B2m^−/−^ mice, in which MHC-I expression is abrogated, or from Ly49-deficient mice. NK cells from these mice exhibited defective cytokine production and impaired recognition and elimination of MHC-I-deficient target cells and were generally hyporesponsive [50,51,52]. A more recent study using specific genetic models showed that NKG2A and inhibitory members of the Ly49 receptor family synergize to regulate NK cell education. Mice lacking all Ly49 receptors showed comparable results to mice lacking only inhibitory Ly49 proteins (Ly49I and Ly49C) in terms of reduced IFNγ production and tumor control. Mice lacking only NKG2A showed similar results but with milder differences in comparison to wild-type mice. Only combined deletion of inhibitory Ly49 receptors and NKG2A showed results comparable to those from B2m^−/−^ mice [53]. Nevertheless, it seems that the process of education is not final but rather ongoing and fluid since environmental changes can alter the licensed state of even fully mature NK cells. Evidence for this comes from the fact that NK cells from MHC-I deficient mice restored their function after adoptive transfer to wild-type mice [54].

In humans, NK cell education also takes place, predominantly using inhibitory receptors from the KIR receptor family and NKG2A [55]. KIR receptors are highly variable and recognize polymorphic determinants of HLA-A, -B, and -C. In contrast, NKG2A is a conserved receptor that recognizes leader peptides derived from classical HLA-A, -B, or -C, presented on the nonclassical class I molecule HLA-E [56]. The HLA and KIR gene families form the most polymorphic receptor-ligand pair in the human genome [57,58]. KIR and HLA genes are located on different chromosomes, allowing them to be inherited independently. As a result, individuals may express KIRs for which the corresponding HLA ligand is absent [59].

During NK cell development, NKG2A is expressed first, while KIRs appear later. CD56^bright^ cells typically exhibit high NKG2A expression and low KIR expression. These cells are weakly cytotoxic but produce high levels of cytokines. As NK cells mature to the CD56^dim^ stage, the expression of KIRs increases [56]. Due to the stochastic nature of inhibitory receptor expression, NK cells can express none, one, or a combination of inhibitory receptors. Cooley et al. demonstrated that CD56^dim^NKG2A^−^KIR^−^ cells have reduced IFNγ production and diminished killing capacity. Conversely, NKG2A^+^KIR^−^ NK cells showed the highest specific lysis against K562 targets, while NKG2A^+^KIR^+^ and NKG2A^−^KIR^+^ cells were comparable. They also showed that CD56^dim^NKG2A^−^KIR^−^ cells are functionally immature but can be induced to proliferate and differentiate in vitro, acquiring KIR and NKG2A receptors and effector functions [56].

NK cells educated by NKG2A/HLA-E develop different responses compared to those educated by KIR. Leijonhufvud et al. observed that despite a positive correlation between KIR-mediated education and CD16 expression, NK cells educated by one or even two inhibitory KIRs did not perform better in terms of ADCC than uneducated NK cells in either missing-self or KIR-ligand matched settings at saturating antibody concentrations. Instead, NKG2A^+^ NK cells consistently showed more potent ADCC in the missing-self context despite lower levels of CD16 expression [60]. Differences between KIR and NKG2A-educated NK cells arise from variations in their cellular metabolism. NKG2A-educated NK cells are more metabolically active and remain more functionally competent when oxidative phosphorylation is restricted [61].

In mice, there is synergy between Ly49 receptors and NKG2A, whereas, in humans, it seems that HLA molecules themselves dictate the relative contributions of KIR and NKG2A to education. Polymorphisms in the HLA leader sequence control the availability of peptides presented by HLA-E to NKG2A. Individuals with KIR-B haplotypes, which are enriched for multiple activating receptors, exhibit greater expression of the inhibitory receptor LIR-1, restoring the balance between activating and inhibitory signals [62]. Thus, human NK cell education is finely tuned at the repertoire level, maintaining a balance between activating and inhibitory effector functions [63].

#### 3.1.2. Education through Activating Receptors

During development, NK cells undergo additional education, which regulates responsiveness to certain activating receptors. In mice, three activating receptors differ from the others expressed in NK cells. These are NKG2D, NKp46 and CD16. These receptors are expressed on all murine NK cells and play an important role in NK cell effector functions. NKG2D and NKp46 recognize induced-self ligands, while CD16 binds the Fc-tail of antibodies and plays an important role in ADCC. Also, there is no inhibitory receptor that recognizes the same ligands. To ensure proper activation through these receptors, their activity is fine-tuned during NK cell development. The developmental process involving NKG2D influences the sensitivity of the NKp46 and CD16 receptors [47]. This regulatory mechanism is mediated through the NKG2D-DAP12 signaling axis, which orchestrates the downregulation of CD3ζ and ZAP-70, which play a role in negatively modulating NKp46 and CD16 signaling. This, in turn, governs the sensitivity of NK cells when encountering cellular targets expressing NKp46 ligands [47]. Mice who lack NKG2D expression, therefore, have dysregulated responsiveness of NKp46 and CD16 receptors and show hyperresponsiveness through these receptors, which results in better control of MCMV infection as well as tumors expressing NKp46 ligands [33,64]. Regrettably, discerning the influence of NKG2D on NK cell development in humans poses a challenge. Currently, no deficiency in this receptor has been observed, and most peripheral NK cells express NKG2D, making it challenging to compare NK cell subpopulations with and without this molecule.

### 3.2. Regulation of NK Cell Responsiveness in the Periphery

In the periphery, NK cell activity is fine-tuned using several mechanisms. In contrast to CD16, NKp46, and NKG2D, many NK activating receptors recognize the same class of ligands as certain inhibitory ones, which are therefore known as paired receptors. Paired receptors can be found in the Ly49 family in mice or the KIR family in humans. Activating receptors in the KIR family are called killer activating receptors (KAR). Their transmembrane sequence contains charged amino acids that interact with an activating adaptor molecule [65]. Ligands of most KARs are unknown, but those of which the ligand is known to recognize MHC class I proteins are also recognized by a KIR inhibitory partner. Inhibitory receptors always show stronger binding to the shared ligand [64]. The question of why we have activating and inhibitory receptors recognizing the same ligands within these receptor families is still unexplained. One of the suggested explanations is that KARs fine-tune the threshold required for both activation and inhibition of NK cells. KIRs exhibit rapid binding and unbinding kinetics with their corresponding MHC class I proteins. Notably, studies have demonstrated that the level of HLA-C proteins influences the effectiveness of NK cell inhibition [66]. Thus, it is plausible that when MHC class I proteins are engaged by KARs, they refine the inhibitory threshold by providing a counterbalancing activating signal [64]. In the Ly49 family, we find one specific receptor pair of Ly49I inhibitory and Ly49H activating receptors. In addition to recognizing MHC class I, Ly49I also recognizes the non-self MCMV encoded protein m157. It is, therefore, believed that this molecule was originally formed by the virus to evade NK cell-mediated control. However, through evolution, the Ly49H activating receptor developed, which now efficiently detects and eliminates MCMV-infected cells in B6 mice [67]. Another example of a receptor pair is DNAM-1 and TIGIT. In the absence of stressors such as infection, signals from the inhibitory receptor TIGIT prevail over those from the activating receptor DNAM-1 since the former has a higher affinity for their common ligand PVR. Upon cellular stress, the expression of PVR is upregulated over a certain threshold at which signals from the activating receptor become dominant [65].

NK cells also express receptors, which have both inhibitory and activating properties, for example, 2B4. The 2B4 receptor belongs to the CD2 family of molecules and is expressed in all human and murine NK cells. The ligand for 2B4 is CD48, which is expressed on all hematopoietic cells and is upregulated during EBV infection [68,69]. Both human and murine 2B4 have four immunotyrosine-based switch motifs (ITSM) in their cytoplasmic tail. For the activating functions of this receptor, binding of the intracellular adaptor molecule SAP (signaling lymphocyte activation molecule (SLAM)-associated protein) is crucial. Evidence for this comes from a study showing that in SAP-deficient humans, 2B4 stimulation leads to a decrease in NK cell lytic activity and IFNγ production [70]. For inhibitory activity, it was thought that EAT-2A and EAT-2B play a role since they can also bind to 2B4 ITSMs [71], but cells lacking expression of these molecules were still able to provide inhibition through the 2B4 receptor [72]. Since both SAP and EAT-2 can bind Fyn, regulation of Fyn kinase recruitment and/or activity may be crucial for regulating activating or inhibitory functions of the 2B4 receptor [73]. It is clear that the regulation of 2B4 activity is complex, and the decision on whether inhibitory or activating signals will be transduced depends on the degree of receptor expression, the extent of its ligation, and the relative abundance of certain adaptor molecules [72].

**Figure 2:**
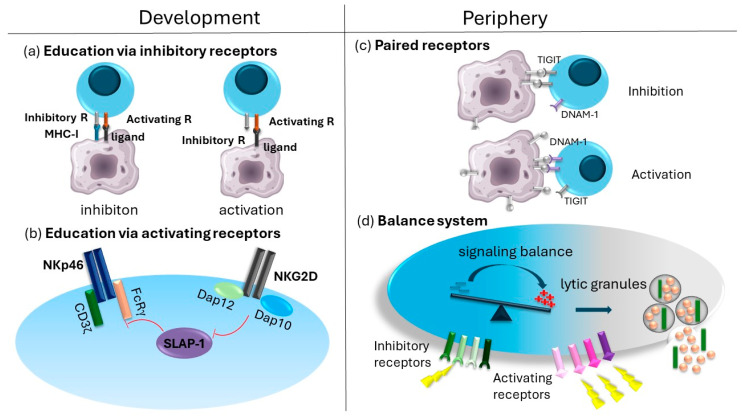
Regulation of NK cell activity: NK cell activity is regulated at multiple levels, both during development and in the periphery. During development, NK cells undergo classical NK cell education (**a**) as well as specific education that establishes a threshold for activating receptors such as NKp46 (**b**). In the periphery, their activity is tightly controlled through mechanisms involving paired receptors (**c**) and is further safeguarded by the balance system (**d**).

In summary, NK cell responsiveness is a carefully regulated process, depending on several educational steps during development, which is further fine-tuned in the periphery. Its ultimate goal is to generate cells that manage an impressive balancing act, retaining sufficient sensitivity to cellular threats while preventing detrimental hyperreactivity (Figure 2).

### 3.3. Tipping the Balance

Recently, the question of whether the balance system is sensitive enough or whether NK cells need an additional signal from a specific receptor to trigger their activation arose. The NKG2D, NKp46, and CD16 receptors are expressed on all murine NK cells, do not have inhibitory counterparts, and share a unique way of fine-tuning their reactivity, posing them as potential candidates. Mice lacking all three of these receptors were generated (Triple knockout mice-TKO mice). Analysis of TKO mice showed that NK cells do not require signals from these receptors to be activated, revealing the remarkable plasticity of NK cell responsiveness. The combined loss of NKG2D, NKp46, and CD16 receptors resulted in a significant reduction in the ability of NK cells to control viral infection and non-hematopoietic tumors. However, their functionality was not abrogated completely [74]. Interestingly, apart from the function of CD16 associated with ADCC, the loss of these receptors was partially compensated by differential expression and sensitivity to activation by other activating receptors [46]. TKO NK cells showed reduced expression of the inhibitory Ly49A and TIGIT receptors while upregulating expression of the activating receptor DNAM-1. Although the expression level of the activating receptor NK1.1 was unchanged, TKO NK cells showed hyperresponsiveness through this receptor [74].

Similar effects were also observed in NKG2D and NKp46 double-knockout mice. NK cells from these mice have increased expression of KLRG1 and DNAM-1 while exhibiting lower expression of Ly49G2 and Ly49F. Although the receptor repertoire was altered, the killing ability of these cells towards different tumor targets remained unchanged. They did, however, exhibit hyperresponsiveness to IL-2 and Ly49D stimulation [75]. Another example is mice lacking all Ly49 receptors. In these mice, there was also an altered receptor repertoire with an increase in the expression of the NK1.1 receptor and a decrease in the expression of NKG2D and DNAM-1 [53].

**Figure 3:**
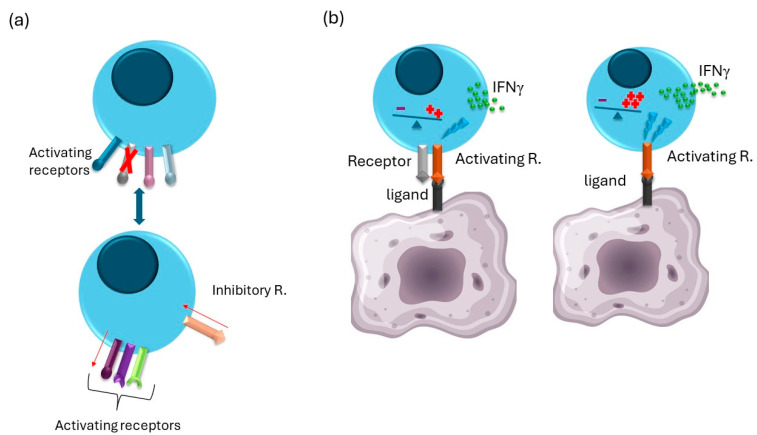
Adaptation of the NK Cell Receptor Profile and Sensitivity Following the Loss of an Activating/Inhibitory Receptor: When an activating receptor is lost, NK cells modify their receptor profile to sustain the “balance system” and preserve their sensitivity. This modification may include the downregulation of some inhibitory receptors and/or the upregulation of particular activating receptors (**a**). Moreover, the loss of one receptor can be offset by the increased responsiveness of another receptor (**b**).

It seems that NK cells compensate for the lack of certain activating receptors by lowering and increasing the expression of inhibitory and activating receptors, respectively, as well as changing threshold levels for the remaining activating receptors (Figure 3). These compensatory mechanisms are already visible in mice lacking only one activating receptor. CD16-deficient NK cells showed hyperreactivity through the NK1.1 receptor [74], while NKp46-deficient NK cells showed hyperreactivity after cytokine stimulation (unpublished data). Similarly, NKG2D deficient NK cells showed higher IFNγ production after stimulation through NKp46 or CD16 receptors [47]. Furthermore, mice lacking CD45 expression showed a decrease in expression of Ly49D and 2B4 receptors [76]. It appears that this effect is not a specific compensatory mechanism for defects in the receptor repertoire, as it is also observed when NK cells lack Fyn kinase, an important downstream activating signaling component. NK cells from Fyn kinase-deficient mice also have changed the expression of certain receptors, such as Ly49A and Ly49D [77]. This shows the remarkable compensatory mechanism of NK cells, allowing them to have similar activation thresholds despite having a different receptor repertoire.

## 4. Memory NK Cells

An additional level of peripheral modulation of NK cell responsiveness occurs in a subset of NK cells after activation. Nearly two decades ago, it was discovered that natural killer (NK) cells can develop memory-like populations capable of mounting a robust recall response. NK cell memory generation occurs following certain viral infections, contact hypersensitivity reactions, and stimulation by pro-inflammatory cytokines. Numerous reviews detail the generation and role of memory NK cells in various infections [78,79,80,81], so we will not elaborate on that here. However, it is important to mention that memory NK cells, compared to naïve cells, show a change in receptor expression and activation threshold.

Although emerging evidence shows that various viral infections such as HIV/SIV, vaccinia virus, and influenza virus can induce memory NK cell formation, its development has been most thoroughly studied in the context of the murine cytomegalovirus (MCMV) infection [80]. In this model, the interaction between the Ly49H receptor and its viral ligand m157 leads to the expansion of a virus/m157-specific NK cell subset. This results in a long-lasting enhanced secondary response, providing improved protection against MCMV compared to naïve NK cells [82]. Memory NK cells show higher expression of Klrg1, CD43, and Ly6C and reduced expression of CD27. Compared to naïve NK cells, memory NK cells exhibit better IFNγ production and degranulation after stimulation through NK1.1, although NK1.1 expression remains unchanged. Memory NK cells also have higher expression of the Ly49H receptor and show a better cytokine response after exposure to m157-expressing target cells [82]. Similar observations were made in cytokine-induced memory NK cells. Cytokine-induced memory NK cells upregulate multiple markers of NK cell activation and maturation following homeostatic expansion and proliferation [83]. In addition, these cells show higher IFNγ production after restimulation with cytokines and after stimulation through NK1.1 or Ly49H [84]. In humans, memory NK cells show several modifications associated with altered activation thresholds. Human memory NK cells have shown a loss of the adapter molecule FcεRIγ, as well as signaling molecules Syk and EAT2 [85,86]. At the same time, higher constitutive levels of IFNγ transcripts have been observed, which were proposed to be due to epigenetic modifications. The NKG2C^+^ NK cell population that expands in HCMV-seropositive individuals exhibits a demethylated CNS1 region in the *Ifng* locus, facilitating transcription [87]. Taken together, these findings indicate that memory NK cells modulate their activation threshold, increasing sensitivity and allowing for a more potent response upon antigen re-encounter.

## 5. Therapeutic Implications of NK Cells

Natural killer (NK) cells, as cytotoxic innate lymphocytes, are promising targets for immunotherapy due to their ability to lyse tumor cells and release proinflammatory cytokines without requiring prior sensitization. Unlike T cells, NK cells are not restricted by human leukocyte antigen (HLA) and can mediate graft-versus-leukemia (or tumor) effects without causing graft-versus-host disease (GvHD). Despite the potential of NK cell immunotherapy, challenges include limited persistence in vivo, poor infiltration into solid tumors, clinical-grade expansion, and tumor editing [88].

NK cell-based immunotherapy can target either inhibitory or activating receptors, but it must aim to shift the NK cell balance system toward activation. In the tumor microenvironment, impaired NK cell function is often linked to increased expression of inhibitory receptors, which can bind to ligands on tumor cells, thus evading immune recognition. Inhibitory receptors include PD-1, LAG3, TIM3, and TIGIT. These receptors can be blocked using antibodies to prevent their inhibitory activity, a strategy known as immune checkpoint therapy. Various inhibitory receptors, including KIR and LIR family members, NKG2A/CD94, TIGIT, LAG-3, TIM-3, and CTLA-4, as well as IL-1R8, are being investigated for immune checkpoint therapy [89,90,91,92,93,94]. The most successful immunotherapy, anti-PD-1/PD-1L treatment, stimulates both T cell and NK cell responses [95,96].

Many NK cell-based immunotherapies focus on enhancing the Chimeric Antigen Receptors (CARs) [97]. CARs are synthetic fusion proteins with an extracellular antigen recognition domain and an intracellular signaling domain that activates the cell. Initially designed for T cells, CAR T cell therapy has shown potential but also presents challenges like severe side effects (cytokine release syndrome and neurotoxicity), autologous donor limitations, and issues with T cell quality and quantity in heavily pre-treated patients [98,99]. This is why NK cells present an attractive alternative. Also, in addition to CAR, NK cells have other activating receptors that can detect ligands on tumor cells and prevent tumor evasion by downregulating CAR targets.

Recent findings indicate that modulating the surface expression of activating receptors triggers changes in the expression of other receptors to maintain the NK cell activation threshold. In CAR NK cells, the overexpression of specific CAR molecules can alter NK cell phenotype or activity. Supporting this hypothesis, transcriptomic analyses have revealed differences in gene expression between CAR NK cells and untreated NK cells, including higher expression of cytotoxic factors and changes in receptors like TIGIT, KLRC1, KLRD1, and NKp44 [100,101]. In vivo engagement also alters the transcriptional profile of NK cells, with differences noted in receptors and signaling molecules such as DNAM-1, NKp30, KIR2DS4, and Zap70 [102]. The CD3ζ molecule is frequently used as an adaptor in CARs. Studies have shown that variations in CD3ζ expression levels affect the activation threshold of other activating receptors [47]. Further research is necessary to determine whether this could hamper the success of CAR NK therapy.

A promising NK cell-based therapy involves NK cell engagers (NKCEs), which trigger an activating receptor on NK cells while simultaneously binding a tumor antigen [103]. This approach is simpler and more affordable than CAR-based therapy and has shown comparable success. NKCEs are synthetic molecules derived from monoclonal antibodies designed to harness NK cell immune functions against cancer. Examples are Bispecific killer cell engagers (BiKEs) and trispecific killer cell engagers (TriKEs), with BiKEs targeting CD16 and tumor antigens, and TriKEs additionally including an IL-15 moiety to enhance NK cell proliferation, activation, and survival [104]. Some TriKEs target multiple tumor antigens simultaneously, such as those targeting CD19, CD22, and CD16 [105]. While a CD16-specific TriKE (and BiKE) primarily takes advantage of ADCC, additional NK cell receptors might also be suitable for NK cell engagers. One candidate is NKG2D since many tumors express NKG2D ligands. The potential downside of the NKG2D receptor is that chronic engagement can cause its downregulation from the cell surface [106]. NKp46 represents another interesting target with the advantage that its expression remains stable in the tumor microenvironment [107].

Recently, a tetraspecific engager was developed containing an IL-2v peptide stimulating IL-2R, an antibody fragment targeting NKp46, an Fc domain of human IgG1 mediating interaction CD16, and an antibody fragment targeting CD20 (aCD20) as a model tumor-associated antigen (TAA) [107]. An IL-2 variant designed with a point mutation that abolishes binding to CD25 was used to limit interaction with Tregs but retains the ability to interact with CD122/CD132 and promote NK cell activation and proliferation [108]. However, whether these molecules cause changes in the activation threshold of NK cells over time is currently unknown.

NKCEs offer a versatile, cost-effective platform that can engage various combinations of activating receptors and novel targeting ligands. However, their use also seems to result in transcriptomic changes affecting receptor and cytotoxic molecule expression. Future research must reveal whether this limits the effectiveness of these compounds in a clinical setting.

## 6. Conclusions

Natural Killer (NK) cells are powerful effectors in antitumor immunity; however, a major challenge in leveraging them therapeutically lies in the incomplete understanding of NK cell activation. Despite extensive research for almost five decades, significant gaps remain in our comprehension of the molecular mechanisms that enable NK cells to target certain cancer cells selectively. Proper regulation of NK cell activation is crucial, as inappropriate activation can result in tissue damage or autoimmune disorders. Currently, there are several therapeutical strategies in development, the most prominent ones being the NK cell engagers and CAR NK cell therapy. Functional plasticity of NK cells suggests that NK cells modulate their phenotype to preserve a certain threshold of activation. Therefore, the deficiency of one or more receptors leads to altered expression of other receptors to maintain the same activation threshold. This poses a new perspective on CAR NK cell therapy, as over-expressing a CAR could lead to altered expression of other receptors present in NK cells and potentially lead to treatment failure. On the other hand, NK cell engagers involve multiple NK cell receptors to try to provide a stronger signal to shift the signal balance toward activation. This could be a novel strategy that does not interfere with the receptor repertoire of NK cells. All of this highlights the need for a deeper insight into the complexity of NK cell biology and elucidating molecular mechanisms underlying NK cell activation. A better understanding of this complex process represents an attractive target for manipulation and more effective use of NK cells in cancer immunotherapy.
